# Demystifying the mirror taboo: A neurocognitive model of viewing self in the mirror

**DOI:** 10.1111/nin.12351

**Published:** 2020-03-27

**Authors:** Wyona M. Freysteinson

**Affiliations:** ^1^ Nelda C. Stark College of Nursing Texas Woman’s University Houston TX USA

**Keywords:** body image, clinical nursing, complementary therapies, disfigurement, mirror, nursing model, trauma

## Abstract

Research has consistently demonstrated that viewing one's body in a mirror after an amputation or other perceived or visible body disfigurements can be a traumatic experience. Mirror viewing or mirroring is a taboo subject, which may be the reason this trauma has not been previously detected or acknowledged. Traumatic mirror viewing may lead to mirror discomfort, mirror avoidance, and a host of psychosocial concerns, including post‐traumatic stress. As mirroring is complex, four qualitative mirror viewing studies, embodiment concepts, polyvagal theory, and memory theories were used to develop a model. In this article, foundational knowledge that led to the development of the model is shared. A neurocognitive model of mirror viewing is offered together with implications for nursing research, practice, and education.

## 1 INTRODUCTION

The act of looking into a mirror each morning and throughout the day is as natural as breathing for many individuals. A mirror is a reflective surface, typically made of glass, and coated with a metal amalgam (mercury and other metals). Reflective surfaces such as windows and shiny objects function as mirrors, albeit not always with a clear image. These objects offer a visual field ordinarily not seen from the standpoint of the first person. When we look into a mirror, the word *mirroring* is an appropriate verb for our actions. We can turn our faces and bodies from side to side, and we can move backward from the mirror and watch our image grow distant. In a sense, we can play with our image. Unlike the permanent image of a photograph, the movement reflected in a mirror is like an instantaneous motion picture. It is only in mirrors and reflective surfaces that we have access to our whole (and broken) bodies, a view that research suggests is equivalent to direct observation of ourselves (Jenkinson & Preston, 2017).

### 1.1 Mirror tools

In psychology, tools that measure body image discomfort include one to two mirror viewing questions. Individuals dissatisfied with their appearance may suffer distress (Carr, Harris, & James, 2000) and feel guilty about their body shape in mirror viewing (Ben‐Tovim & Walker, 1991; Castonguay, Sabiston, Crocker, & Mack, 2014). People who have eating disorders may suffer shame (Duarte, Pinto‐Gouveia, Ferreira, & Batista, 2015) and other negative feelings (Cooper, Taylor, Cooper, & Fairburn, 1987) in viewing their self in a mirror. Individuals who perceive they have an undesirable body image may engage in frequent mirror checking (Brown, Cash, & Mikulka, 1990) or use a mirror to camouflage, in the hope it will make them feel better about their body image (Veale & Riley, 2001). Patients with burns may have difficulty in deciding to look in a mirror (Shepard, Tattersall, & Buchanan, 2014). Individuals with body dysmorphic disorder (Wilhelm, Greenberg, Rosenfield, Kasarskis, & Blashill, 2016), amputations (Gallagher, Horgan, Franchignoni, Giordano, & MacLachlan, 2007), and burns (Shepard et al., 2014) may also avoid mirrors.

### 1.2 Mirror interventions

In rehabilitation, therapists use mirror therapy to reduce pain or improve the function of a limb. Therapists place a mirror to superimpose the unaffected limb in such a way that it appears to be the affected limb. Moving the unaffected limb retrains the affected limb via mirror neuron pathways in the brain (Thieme et al., 2018). The use of mirror training to improve limb strength and performance in healthy individuals is inconclusive (Chen, Wang, Bai, & Wang, 2019). Psychologists have used guided mirror image exposure for individuals with eating disorders and body image disturbances. The theories supporting this intervention suggest exposure acts as counterconditioning or cognitive dissonance and suggest individuals can learn to interpret their bodies positively (Griffen, Naumann, & Hildebrandt, 2018). Researchers (Petrocchi, Ottaviani, & Couyoumdjian, 2016) have found that self‐compassion statements stated in front of a mirror had a significantly positive soothing response and effect on heart rate variability.

### 1.3 Mirror trauma

*Mirror trauma* is shock or severe discomfort in seeing one's body in a mirror, often experienced when seeing or perceiving a dramatic change in one's body. The story that inspired this research trajectory began in 1994 when Freysteinson asked terminally ill women to share a story about viewing themselves in a mirror. Mary (pseudonym) explained that she had a mastectomy five years previously. After three weeks in the hospital, the first thing she did when she arrived home was to go to her bathroom, take off her blouse, and look in the full‐length mirror.

I felt like running out on the road and screaming. Nobody will know what I went through. It was a terrible mess…All this raw stuff hanging there. I thought this is it for me, seeing my body all chopped up like that (p. 126).

Mary's story continued for two paragraphs of text, as she described in vivid detail her mastectomy site and her thoughts and feelings. Researcher notes indicated that Mary's graceful demeanor changed to great anxiety as she told her story in an increasingly louder and faster tone, suggesting post‐traumatic stress disorder (PTSD). Participants (*n = *12) in a study of viewing self in the mirror after a mastectomy (Freysteinson et al., 2012) described similar emotions of shock, disgust, and self‐revulsion in mirroring. Two participants in this study were terrified to look in a mirror and avoided mirrors for days.

In a qualitative study (Freysteinson, Thomas, Sebastian‐Deutsch, et al., 2017), the participants (*n = *17) described viewing self in a full‐length mirror post‐amputation as more distressing than looking down at a missing limb. They discussed how they could disassociate themselves from their bodies when they looked down at missing limbs or viewed their incisional sites in a small mirror. Seeing one's whole self in a full‐length mirror was described as devastating. Some participants discussed feelings of shock, numbness, and a lack of emotion in initial full‐length mirror viewings. For George and Danielle (pseudonyms), who had suffered lower‐limb amputations, the shock was so profound that initially, they were frozen and had no other emotions. Danielle said: ‘It was so surreal that I was not feeling anything’ (pp. 25–26). Participants indicated that the only full‐length mirrors in the hospitals were in public areas: elevators, lobbies, and physical therapy departments. Small mirrors to view the incision sites were non‐existent. In addition to a lack of mirrors, the participants perceived that nurses and other healthcare personnel seemed indifferent and lacked knowledge as to how difficult the mirror viewing experience was post‐amputation.

Patients are often unaware of where full‐length mirrors are in a hospital environment and find themselves accidentally seeing themselves in these public mirrors and other large reflective surfaces after an amputation. The event has been reported as being extremely shocking and is a combination of seeing the reflection of their changed bodies for the first time (Freysteinson, Thomas, Sebastian‐Deutsch, et al., 2017) and the *shopping mall mirror effect.* This well‐known phenomenon occurs when individuals confront another person in a public space, only to discover that the individual is one's reflection (Brandl, 2018; Keenan, Gallup, & Falk, 2003). This shocking experience can leave individuals feeling visibly shaken and uneasy or distressed as to who may have seen their reaction.

Participants in focus groups (Freysteinson, Mellott, et al., 2018; Freysteinson, Thomas, Sebastian‐Deutsch, et al., 2017) were heartened to find others who had been or were going through difficult mirror viewing experiences. The perception of vanity, shame, stigma, and secrecy associated with mirrors had prevented them from previously sharing these events. These distressing feelings are not typical everyday conversations. Conversations integrating a person's own body and mirrors are essentially *taboo.*

### 1.4 Taboo

A taboo is passed on from generation to generation and is a phenomenon that is uncanny, sacred, and forbidden, and it may be associated with magic or sorcery (Levine, 1986). The mirror meets this criterion. The history of the mirror, which includes evil spirits, magic, and bad luck, is far too elaborative to include in this article, however, what is important to this discussion is the notion that for generations mirroring has been considered a *vanity.* In the mythical tale of Narcissus, a young man is so enamored with his reflection in a pool of still water, that he falls into the water and sadly drowns (Ovid, 1955). In the middle ages, some churches considered mirrors the devil's tool (Melchior‐Bonnet, 2002; Pendergrast, 2003). Many children learn the association between vanity and mirrors at an early age. For example, in the Stanford Libraries (n.d.), the fairy tale of the narcissistic queen and her talking mirror in *Snow White and the Seven Dwarfs* logs 62 books, 23 musical recordings, five videos, and 4,723 articles. The popular phenomenon of taking and posting selfies on social media has been called a merging of mirror viewing and bodies in photographs (Tiidenberg & Cruz, 2015). The difference, however, is that one can edit, enhance the image with filters, and choose the best selfie to post.

A key reason for the development of this model is to demystify the mirror taboo and describe and explain the mechanisms of mirroring. This article discusses the foundational knowledge, the model, and implications for practice, research, and education.

## 2 FOUNDATIONAL KNOWLEDGE

Qualitative studies have provided key elements in understanding the phenomenology of mirroring (Freysteinson, 2019). Ricoeur's three key constructs of the phenomenology of the will (Ricoeur, 1966) acted as a heuristic: decision, action, and consent.

### 2.1 Phenomenology of mirroring

Deciding to look at self in a mirror after a change in one's body stems from a motive of curiosity. This motive is often paradoxical: There is a wanting but not wanting to view self. Before looking into a mirror, one anticipates and forms a picture in the mind's eye as to what will unfold in front of the mirror. Looking into a mirror after suffering visible bodily disfigurement may be a moment of profound immobilizing shock. For those not in shock, mirroring is a moment of understanding and evaluation. There is an initial understanding formed by the lens through which one views the world, together with an evaluation, a personal moment that makes sense of the reflection. This evaluation may be medically accurate (i.e., I had radiation, so it is red) or not (i.e., the scar looks like this because I sinned). Consent is lived on a continuum of hope to despair. ‘Consent which reaffirms an existence which is not chosen, with its constriction, its shadows, its contingence, is like a “choice” of myself, a necessary choice’ (Ricoeur, 1966, p. 484).

### 2.2 Embodiment

The concept of embodiment, the lived subjective experience of being one's body (Marchetti, Piredda, & De Marinis, 2016; Mason, 2014; Merleau‐Ponty, 2012), provided an understanding of mirror viewing comfort and discomfort. When there is no disease or trauma, we often are not aware of our bodies. We can get out of bed, go to our bathrooms, and perform our bodily routines in a taken‐for‐granted way. There are moments where we choose to consciously assess our bodies by looking down at, for example, how a watch looks on our wrist or by viewing one's image in a mirror. Following that assessment, we move back into a taken‐for‐granted physical state of being. In a grounded theory study, Piran (2016) studied the experience of embodiment and uncovered positive and negative poles of embodiment. One pole was ‘body connection and comfort versus disrupted body connection and discomfort’ (p. 49). When one is comfortable with one's body, one feels as one or at home with one's body. When there is an illness, injury, pain, or trauma, ‘the way in which the body is thrown out of taken‐for‐grantedness and into full consciousness can be extreme’ (Draper, 2014, p. 2237). Actions that were at one‐time effortless (i.e., mirror viewing) can shock and confuse us.

### 2.3 Shock

Porges’ (2018) polyvagal theory of the autonomic nervous system added insight into the shocking, immobilizing mirror reactions that participants have reported. The theory postulates that the autonomic nervous system has two defensive pathways: fight or flight and immobilization. In theory, these two adaptive neural pathways work outside of conscious awareness. The immobilizing polyvagal reaction is an involuntary neurological response to a dangerous or life‐threatening situation in the environment. In this state, individuals may experience detachment or dissociation from their bodies. In fight or flight, there is distress, feelings of being overwhelmed, and strong emotions. Also, anticipating one's reflection after bodily disfigurement after an amputation or mastectomy can trigger images in one's mind that are so terrifying that there is a fright/flight or immobilizing polyvagal reaction. A polyvagal reaction can trigger an increased heart rhythm or can trigger diminished heart rate variability, and a host of physiological and psychosocial disorders, including PTSD (Gordon, 2019; Porges, 2018).

One perplexing question that came out of the research (Freysteinson, Thomas, Sebastian‐Deutsch, et al., 2017) was: Why is it that, post‐amputation, a person can look down at a missing leg for days and then suffer severe shock (immobility) when viewing self in a full‐length mirror? This question led to an investigation into the literature of self‐awareness and self‐recognition.

### 2.4 Self‐recognition

The psychological theory of objective self‐awareness (Duval & Wicklund, 1972; Wicklund, 1975) suggests that when looking in a mirror, one of two responses occur. When self‐evaluation is positive, this is a positive motivation, and one will actively seek out and use mirrors. When self‐evaluation is negative, one will act to reduce a negative discrepancy between the ideal and real self or actively avoid the mirror. Later research demonstrated that self‐awareness differs in individuals with distinct cognitive, social, motivational, and behavioral effects (Carver & Scheier, 1981).

Some psychologists have theorized that there is a correlation between self‐awareness and self‐recognition (Keenan et al., 2003). Morin (2006) challenged this viewpoint and suggested that there was no discernible link between self‐awareness and self‐recognition. He concluded that to recognize oneself in a mirror may be due to ‘a lower form of self‐awareness, or a higher level of consciousness ‐ that is, phenomenal self‐acquaintance’ (p. 367).

A review (Devue & Bredart, 2011) of 18 neuroimaging studies suggested that self‐recognition was a complex neurological network involving primarily the right frontal and parietal areas of the brain. These studies used pictures, morphed pictures, and movies to detect self‐recognition. A meta‐analysis (van Veluw & Chnace, 2014) of seven fMRI studies of self‐recognition using pictures found the brain areas that activated in self‐face recognition were in the right superior temporal parahippocampal, anterior cingulate cortex/inferior frontal gyrus, and the left inferior parietal lobe (*p*‐value < 0.05). These literature reviews do not necessarily mean that self‐recognition using a mirror occurs in these areas of the brain. However, this review does provide a strong possibility that mirroring activates an area in the prefrontal cortex and parietal lobe.

### 2.5 Memory

A question that has plagued the author throughout this research trajectory has been: Why are some mirror images locked in memory and can be vividly described years later, and yet one cannot remember other images? Typically, people cannot recall or describe their mirror image reflections at a young age and have only a fleeting image of their reflection earlier in the day. A review of memory theories (Camina & Guell, 2017; Crespo & Fernandez‐Lansac, 2016) provided the final and key aspects of this proposed neurocognitive model of viewing self in the mirror. Camina and Guell (2017) synthesized the theories of memory into one coherent theory. This model uses elements from that theoretical synthesis.

## 3 MIRROR VIEWING MODEL

The following synopsis of the model provides the overarching aspects of mirroring and supportive measures.

### 3.1 Synopsis

The act of seeing one's reflection in a mirror is primarily a neurocognitive function. For many individuals, there is an experience of ease or comfort when performing daily routines in a mirror. The mind registers a consistent self‐recognition. However, when one experiences a visible physical disfigurement, such as an amputation, the mind does not register self‐recognition. Instead, there is a negative disruption or discharge of the neural network of short‐term memory. This disruption leads to a negative mirror viewing experience ranging from discomfort to *mirror trauma*, where there is a significant polyvagal response. Supportive measures may mitigate mirror trauma. Without supportive measures, the trauma of viewing self in a mirror after amputation may lead to adverse psychosocial indicators for some individuals, including ongoing mirror trauma, mirror avoidance, and post‐traumatic stress. Supportive measures and viewing one's new image in a mirror over time may reconfigure the memory to the self‐recognition state and return the mirror viewing experience to a state of ease or comfort.

### 3.2 Neurocognitive mirror model

Seeing one's reflection in a mirror or mirroring is a neurocognitive function (see Table 1 for definitions). Psychosocial emotions (i.e., self‐esteem, shame, guilt) may act as mediators or moderators of variables. In habitual mirror viewing, when an individual is experiencing complete mirror comfort, mirrors are merely tools that act as an extension of the body as do, for example, clothes and watches. When looking into a mirror, the visual sensory information is taken up through the vision system's rods and cones, ganglionic cells, and occipital lobe via the iconic sensory register, a short‐term storehouse where post‐categorical information is compared with the new visual information within milliseconds. When the image is identified, the information transfers to the short‐term memory where its meaning is quickly realized and dissipates (Camina & Guell, 2017). Bodily routines are performed in front of a mirror with ease. In one mirror session of combing one's hair, hundreds to thousands of these images are being taken up by the sensory register. A sliver of this visual information is retained in the short‐term memory and processed for retrieval, providing a persistent or consistent *mirror memory*, in which one recognizes one's self‐image in a mirror from one viewing episode to another. This habitual mirror viewing is so taken for granted that small scars and wrinkles on one's face and body may not even be noted.

**TABLE 1:** Mirror viewing definitions

Mirror Comfort: The degree of comfort/ease one has in viewing and evaluating one's appearance (whole‐body) in a mirror
Mirror Discomfort: The degree of discomfort/stress one has in viewing and evaluating one's appearance (whole‐body) in a mirror
Mirror Trauma: When viewing a radical or perceived change in one's body, there is a disruption of the neural pathways in the frontal cortex, leading to a polyvagal sympathetic nervous system response
Mirror avoidance: The fear of and a conscious decision to avoid looking at one's body or a part of one's body image in mirrors. When suddenly faced with a mirror (as in a department store) or a highly reflective window, one quickly averts one's eyes as the image may be seen as repulsive

A slight change in bodily appearance (i.e., removal of a mustache, a new hair color, or cold sore) or a conscious decision to scrutinize one's body or an area of one's body in the mirror creates a mild disruption in the iconic sensory register and short‐term memory in that the visual information is foreign. The short‐term image (i.e., an image of oneself with orange hair) may enter the long‐term memory.

Mild to extreme discomfort is experienced in mirroring. When this disruption occurs, there is a natural cognitive inclination to view self in the mirror more often for approximately two to three days, which neurologically works to process and solidify a new mirror memory resulting in mirror comfort in subsequent viewing episodes.

When there is a drastic change to one's body, as in an amputation, the visual information is taken up by the iconic sensory system; however, there is a massive disruption to the neurological network in the prefrontal cortex short‐term memory as the new image is incompatible with the mirror memory. This discordant image results in mirror trauma, which is a swift polyvagal response of fright/flight or complete shutdown and atypical heart rate variability. In shutdown or an immobilized state, there is an immediate risk of fainting. These responses are experienced as an internal *jolt* within the body and can be perceived as incomprehensible, frightening, and distressing, and perhaps one of the reasons why mirrors are on the taboo list. For individuals who have this bodily sensation in public, there may be an aftermath of panic or fear that others may have noted the reaction. These polyvagal responses can lead to a host of physiological and psychological disorders. The traumatic mirror image memory houses in the long‐term memory where it is retained indefinitely.

The long‐term memory retains associative and non‐associative memories. Of interest is the non‐associative memory storage house, as it is believed to be responsible for the new behaviors one learns through isolated episodes (Camina & Guell, 2017). In habituation, acetylcholine is consumed with each episode in that the effectiveness of the stimulus (i.e., mirror image) is decreased, which in this model eventually results in habitual mirror viewing. In other words, repeated mirror viewings or exposure restores the mirroring experience to comfort over time. There is not a gradual direct slide into comfort, rather it is perceived as fits and starts. In other words, one may be able to use the mirror more comfortably one time, and in the next episode, there may be a negative reaction. Feelings of anguish and self‐revulsion may accompany some mirror viewings. This alteration between negative and positive experiences (and potentially varied amounts of uptake of acetylcholine) may progressively lead to ongoing mirror viewing comfort.

Individuals who suffer traumatic‐viewing episodes may have difficulty in achieving or ever having mirror comfort. The opposite of habituation is sensitization (Camina & Guell, 2017), which is due to an uptake of serotonin and excess acetylcholine secreted, resulting in a mirror trauma each time one is confronted with a mirror image of self. Mirror avoidance is, for these individuals, the only self‐perceived cognitive approach to avoiding this response. The traumatic mirror memory that was retained in the long‐term memory can become intrusive, which is, together with mirror avoidance, characteristic of PTSD (Crespo & Fernandez‐Lansac, 2016; Gordon, 2019). The primary goal of any intervention is to mitigate the autonomic polyvagal response. Recommendations for interventions are described below in implications for practice.

For some individuals who have had a visible body disfigurement, anticipatory fear, and an accompanying fright/flight polyvagal response may occur before the initial mirror viewings. The reasons for this fear are unique to each individual. Participants (Freysteinson, Mellott, et al., 2018; Freysteinson, Thomas, Sebastian‐Deutsch, et al., 2017) have suggested searching for images of similar disfigurements or having seen a person with a similar disfigurement has led to this fear. Ironically, some individuals have found that the image that was in their mind was far worse than the mirror viewing, resulting in mild mirror discomfort.

An underlying assumption in this model is that individuals who are comfortable viewing self in the mirror will be more comfortable in society and intimacy. In a video developed to teach nurses and other healthcare professionals how to assist individuals in mirroring after an amputation, eight actors with amputations chose to use their own stories to highlight the key talking points of the trajectory of viewing self in a mirror after an amputation. One young man said:

Once you accept yourself in that mirror, everybody else accepts you. Once you have that confidence in yourself, it’s okay. You look in the mirror today, and you step out in the world and everybody sees it. They don’t see your prosthetic or anything you are missing anymore. They are just looking at you (Freysteinson, Thomas, Thayer, Lelek, & Lee, 2018, 13:01).

## 4 IMPLICATIONS FOR NURSING

### 4.1 Practice

Healthcare environments are extremely busy: It may seem easier to leave patients to their own devices in mirror viewing. This practice would be a grave disservice to patients, as the outcomes are that mirrors are eventually faced alone or by surprise, triggering a polyvagal response, the result of which has been noted above: mirror avoidance, ongoing mirror trauma, and psychological pathology including PTSD. It is beyond the scope of this article to describe the psychological and physical effects of trauma (see Gordon, 2019).

Fulton, Mayo, Walker, and Urden’s (2019) model provides a process for situating mirror interventions in practice. ‘Beginning with the end in sight’ (p. 514), the overarching goal is to mitigate mirror trauma and subsequent psychological and physical fallout. The immediate need for patients who have suffered an amputation or other disfiguring event is to *feel safe* (Porges, 2018).

Resources needed are mirrors, mirror education for nurses and other healthcare employees, and a strategy to roll out sensitive and emotional interventions. Small hand‐held mirrors for viewing incisions in preparation for self‐care and full‐length mirrors, especially for those patients with lower body disfigurement, are needed. As many hospitals do not have full‐length mirrors in the patient rooms, hang full‐length mirrors, or purchase rolling portable mirrors. Mirrors must be offered as people do not ask for these tools due to the unstated vanity and taboo that is associated with mirrors (Freysteinson, Thomas, Sebastian‐Deutsch, et al., 2017; Freysteinson, Thomas, Thayer, Lelek, & Lee, 2017; Freysteinson, Thomas, et al., 2018).

Initial mirror viewings must not be seen as a task to be *checked off* on a list. This sensitive intervention cannot be delegated to an aide. Nurses, with their loving bedside presence, soft tone of voice, and listening skills, are in an ideal position to provide education, offer mirrors, and help a patient feel safe (Theede, 2018). A video has been prepared that may help to improve nurses’ confidence in assisting patients in mirror viewing after amputation (Freysteinson, Thomas, et al., 2018). In addition to the video, manager–nurse discussions are needed to allay nurses’ fears (Thayer et al., in press).

Strategies need to be tailored to individuals’ needs. Assess patients for readiness for mirror viewing. The ideal state for a patient to be in is an autonomic ‘window of tolerance’ (Porges, 2018, p. 62), a state commonly known as a parasympathetic state: The pulse and respirations are within normal limits, and an individual appears calm. Patients who are in distress due to pain or severe anxiety require appropriate medications.

Studies (Freysteinson, 1994; Freysteinson et al., 2012; Freysteinson, Thomas, Sebastian‐Deutsch, et al., 2017) have found that patients will, in secret, remove their dressings to see an incisional site in front of a mirror for better visualization. This private covert action is due to the taboo associated with mirrors and vanity. Initially, consider offering a small mirror to view the incisional site. A full‐length mirror viewing is essential as it is in these mirrors that mirror trauma with its accompanying polyvagal response of fright/flight or immobilization often occurs in public places (i.e., hospital elevators, lobbies).

Educate patients hours before mirror viewing, giving them time to decide to view themselves in a mirror later in the day. The goal of education is to demystify mirrors and prepare the patient for the mirror viewing experience. Explain that viewing self in a mirror after visible bodily disfigurement may cause a normal disruption of the mirror memory, the memory housed in our brains from previous mirror viewing episodes. This disruption can lead to involuntary feelings of shock, numbness, devastation, and a host of other emotions, all of which are *normal*. Patients should be encouraged that the mirror memory will, in time, return to a normal state with mirror viewing. For further guidance, a video is available for new amputees developing by eight amputees (Freysteinson, Thomas, Thayer, et al., 2017): Offer patients this video as part of the pre‐mirror or post‐op education. Ask patients to think about who they want in the room (i.e., nurse, loved one) or if they prefer privacy during the mirror session.

Before introducing the mirror, the patient must be in a safe position to prevent falls. Ensure the environment is calm and quiet during the intervention. Soft lighting, as opposed to stark light, is recommended. Have the patient take some deep breaths, short inhalations followed by long exhalations. These procedures help to ensure the patient is in a calm state before mirror viewing. During mirror viewing, provide silence and a loving presence as a patient initially views what may be a forever changed body. A gentle may help to soothe and ground patients (Theede, 2018). Allow patients to view themselves in mirrors as often as they would like to. Upon discharge, educate patients to use a small mirror, if appropriate, to aid in visualizing incision site self‐care. Patients should be encouraged to continue looking into a full‐length mirror daily or more often to return the mirror memory to a *new normal* and mirror viewing to a state of ease or comfort. Encourage patients to take deep, relaxing breaths before and during mirror viewing at home.

**FIGURE 1:**
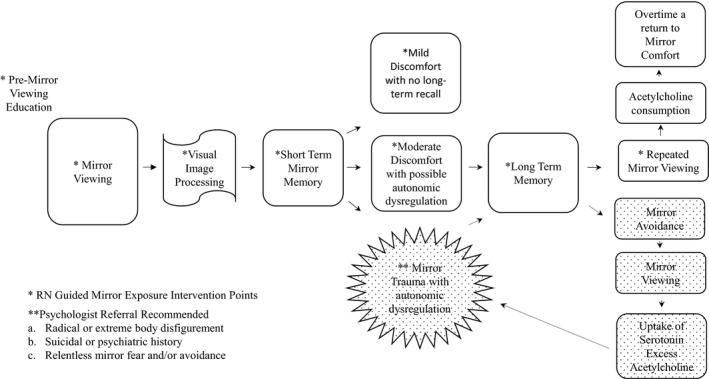
Neurocognitive model of mirror viewing with guided mirror exposure intervention points

In cases of severe body mutilation, as in the loss of several limbs or severe burns, or in individuals who have a known suicidal or psychiatric history, a psychologist should be involved before and if possible in the initial mirror viewing(s). Also, consult a psychologist in cases of complete fear of viewing self in a mirror and persistent mirror avoidance. This consultation must be done in a hospital as a patient will pass by mirrors or highly reflective surfaces in the hospital corridors, home, and/or en route to a psychologist's office (see Figure 1 for nursing and psychologist intervention points).

Physicians, chaplains, therapists, aides, and other healthcare personnel require mirror education in order to understand why nurses are using mirrors with their patients. Therapists who perform activities with patients in front of mirrors need to know if patients have seen their visible body changes in a mirror, to avoid having a surprise first viewing in a public therapy department. Nursing aides who provide personal hygiene and toileting care need to request professional nursing assistance when patients with visible body injuries that may encounter a mirror (i.e., in the restroom) that will allow them to see the disfigurement. Healthcare personnel who transfer patients by wheelchair within the hospital need to know where full‐length mirrors are (i.e., some elevators and lobbies). Patients in wheelchairs who have disfiguring visible injuries should not be pushed into areas when they can view themselves in these mirrors. Alternatively, educate wheelchair porters on how to gently warn patients of mirrors that cannot be avoided (i.e., in elevators).

Continue to monitor and evaluate the mirror intervention implementation process. Assess mirror resources for adequacy. Consider, for example, do rolling full‐length mirrors work for this intervention, or should full‐length mirrors be considered for patient rooms? Evaluate the acceptability and feasibility of the intervention through discussions with patients, nurses, and other healthcare personnel. Ask for patient's opinions of the intervention and monitor satisfaction reports. As with all processes, there will be ways in which to improve this intervention.

### 4.2 Research

Interventional studies are needed to determine if mirror trauma can be effectively mitigated for individuals who have suffered a bodily disfigurement and if those interventions may have positive long‐term psychological effects. Research is needed to determine the best mirror practices for protocol development for all populations (i.e., amputation, mastectomy, burn, paralysis). In a similar vein, continued research is needed for interventions for those individuals who currently suffer severe mirror discomfort or mirror avoidance.

Research is needed to understand why women veterans who have suffered military sexual trauma avoid mirrors or see ‘a stranger’ (Freysteinson, Mellott, et al., 2018, p. 5) when viewing themselves in a mirror. Correlational studies are needed to discern if mirror comfort/avoidance is associated with one's perception of their societal and intimacy body images. A research tool that measures the value of mirror interventions is needed (one such tool measuring mirror comfort and mirror avoidance is currently undergoing validation studies). Research is needed to determine the precise neurological pathways of mirroring. Knowledge is needed to understand the mirror experience for populations that may have visual perception challenges (i.e., autism, dementia).

Considering the polyvagal effect that can occur with mirroring after visible disfigurement, a study of the number of falls in hospital restrooms or other areas that have mirrors, for those who have had surgery or trauma that caused significant incisions and/or visible disfigurement, would be of interest in determining if mirror viewing was a factor. Likewise, research as to whether full‐length mirrors may aide in decreasing falls after lower‐limb amputation, by increasing conscious thought that a lower limb is missing. Although vaguely related to the premise of this theory, a study of the use of small mirrors in self‐care of incisions and prosthetic sites in reducing readmissions due to infection may be significant.

### 4.3 Education

Senior nursing students are often overwhelmed with the massive amount of information they need to retain and critically apply in the clinical area. We must teach students to focus on physiological assessment, technical skills, and the pharmacological aspects of nursing. Students frequently consider the psychosocial aspect of care as being another body system. However, students must learn to care for patients holistically. This dual focus is incongruent (Freysteinson, 2019). To transcend this dialectic for undergraduate nursing students, consider telling students that the goal of nursing is to keep patients in a calm and healing autonomic physiological state. When student nurses learn that a calm autonomic state makes blood draws and inserting intravenous catheters and other tubes into patients’ bodies easier, there is a eureka moment. One fundamental key is that in addition to keeping patients safe, nurses need to help patients *feel safe*. Patients feel safe when their call lights quickly answered, and pain medications are given when needed. Antianxiety medications may be required. When medicine is exhausted, nurses can influence an autonomic healing state through their loving presence, calm tone of voice, listening, touch, soft light, a calm, quiet environment, and a reduction of trauma, including mirror trauma.

## 5 CONCLUSION

The science of mirror viewing is complex. The model proposed in this article suggests that mirroring is primarily a neurocognitive process within the prefrontal cortex and parietal lobe. The memory and autonomic nervous system are key physiological elements. The experience of viewing self in the mirror can be understood from a phenomenological and embodiment point of view. The possibilities for research in trauma, body image, and infection and fall rates are vast. The implications practice section has provided a basic foundation on which to refine and build future mirror viewing protocols. The autonomic nervous system may be central to helping student nurses understand the need for the art of nursing, including mitigating mirror trauma for those who have suffered a visible disfigurement. Mirror trauma is one of the few traumas that healthcare professionals have the power and potential to mitigate or eliminate.

## References

[nin12351-bib-0001] Ben‐Tovim, D. I. , & Walker, M. K. (1991). The development of the Ben‐Tovin Walker body attitudes questionnaire (BAQ), a new measure of women's attitudes toward their bodies. Psychological Medicine, 21, 775–784. 10.1017/S0033291700022406 1946865

[nin12351-bib-0002] Brandl, J. L. (2018). The puzzle of self‐recognition. Phenomenology and the Cognitive Sciences, 17, 279–304. 10.1007/s11097-016-9486-7

[nin12351-bib-0003] Brown, T. A. , Cash, T. F. , & Mikulka, P. J. (1990). Attitudinal body‐image assessment: Factor analysis of the body‐self relations questionnaire. Journal of Personality Assessment, 55(1–2), 135–144. 10.1080/00223891.1990.9674053 2231236

[nin12351-bib-0004] Camina, E. , & Guell, F. (2017). The neuroanatomical, neurophysiological and psychological basis of memory: Current models and their origins. Frontiers in Pharmacology, 10.3389/fphar.2017.00438 PMC5491610 28713278

[nin12351-bib-0005] Carr, T. , Harris, D. , & James, C. (2000). The Derriford appearance scale (DAS‐59): A new scale to measure individuals' responses to living with problems of appearance. British Journal of Health Psychology, 5, 201–205. 10.1348/135910705X27613 15969855

[nin12351-bib-0006] Carver, C. S. , & Scheier, M. F. (1981). Attention and self‐regulation: A control‐theory approach to human behavior. New York, NY: Springer‐ Verlag.

[nin12351-bib-0007] Castonguay, A. L. , Sabiston, C. M. , Crocker, P. R. E. , & Mack, D. E. (2014). Development and validation of the body and appearance self‐conscious emotions scale (BASES). Body Image, 11, 126–136. 10.1016/j.bodyim.2013.12.006 24548436

[nin12351-bib-0008] Chen, Y. , Wang, P. , Bai, Y. , & Wang, Y. (2019). Effects of mirror training on motor performance in healthy individuals: A systematic review and meta‐analysis. BMJ Open Sport & Exercise Medicine, 5, e000590 10.1136/bmjsem-2019-000590 PMC6937065 31908833

[nin12351-bib-0009] Cooper, P. J. , Taylor, M. J. , Cooper, Z. , & Fairburn, C. G. (1987). The development and validation of the body shape questionnaire. International Journal of Eating Disorders, 6(4), 485–494. 10.1002/1098-108X(198707)6:4<485:AID-EAT2260060405>3.0.CO;2-O

[nin12351-bib-0010] Crespo, M. , & Fernandez‐Lansac, V. (2016). Memory and narrative of traumatic events: A literature review. Psychological Trauma, 8(2), 149–156. 10.1037/tra0000041 25915647

[nin12351-bib-0011] Devue, C. , & Bredart, S. (2011). The neural correlates of visual self‐recognition. Consciousness and Cognition, 20, 40–51. 10.1016/j.concog.2010.09.007 20880722

[nin12351-bib-0012] Draper, J. (2014). Embodied practice: Rediscovering the ‘heart’ of nursing. Journal of Advanced Nursing, 70(10), 2235–2244. 10.1111/jan.12406 24673656

[nin12351-bib-0013] Duarte, C. , Pinto‐Gouveia, J. , Ferreira, C. , & Batista, D. (2015). Body image as a source of shame: A new measure for the assessment of the multifaceted nature of body image shame. Clinical Psychology & Psychotherapy, 22(6), 656–666. 10.1002/cpp.1925 25316416

[nin12351-bib-0014] Duval, S. , & Wicklund, R. A. (1972). A theory of objective self‐awareness. New York, NY: Academic Press.

[nin12351-bib-0015] Freysteinson, W. M. (1994). Mirroring: The lived experience of viewing self in the mirror for terminally ill women. Unpublished Master’s Thesis, University of Saskatchewan, Saskatoon, Saskatchewan.

[nin12351-bib-0016] Freysteinson, W. M. (2019). A synopsis of Ricoeur’s phenomenology of the will: Implications for nursing practice, research, and education. Journal of Holistic Nursing, 37(1), 87–93. 10.1177/0898010118778904 29897017

[nin12351-bib-0017] Freysteinson, W. M. , Deutsch, A. S. , Lewis, C. , Sisk, A. , Wuest, L. , & Cesario, S. K. (2012). The experience of viewing self in the mirror after a mastectomy. Oncology Nursing Forum, 39(4), 361–369. 22750894 10.1188/12.ONF.361-369

[nin12351-bib-0018] Freysteinson, W. M. , Mellott, S. , Celia, T. , Du, J. , Goff, M. , Plescher, T. , & Allam, Z. (2018). Body image perceptions of women veterans with military sexual trauma. Issues in Mental Health Nursing, 39(8), 623–632. 10.1080/01612840.2018.1445327 29648911

[nin12351-bib-0019] Freysteinson, W. M. , Thomas, L. , Sebastian‐Deutsch, A. , Douglas, D. , Melton, D. , Celia, T. , … Bowyer, P. (2017). A study of the amputee experience of viewing self in a mirror. Rehabilitation Nursing, 2(1), 22–32. 10.1002/rnj.256 PMC5228627 26879100

[nin12351-bib-0020] Freysteinson, W. M. , Thomas, L. , Thayer, B. V. , Lelek, N. , & Lee, I. (2017). Reflections of healing: Viewing self in the mirror after an amputation. Produced by Texas Woman’s University. https://www.youtube.com/channel/UCRmul9Eqw_HUgcv97on6H8g

[nin12351-bib-0021] Freysteinson, W. M. , Thomas, L. , Thayer, B. V. , Lelek, N. , & Lee, I. (2018). Assisting individuals to view self in the mirror after an amputation. Produced by Texas Woman’s University. http://www.youtube.com/channel/UCRmul9Eqw_HUgcv97on6H8g

[nin12351-bib-0022] Fulton, J. S. , Mayo, A. , Walker, J. , & Urden, L. D. (2019). Description of work processes used by clinical nurse specialists to improve patient outcomes. Nursing Outlook, 67, 511–522. 10.1016/j.outlook.2019.03.001 31030905

[nin12351-bib-0023] Gallagher, P. , Horgan, O. , Franchignoni, F. , Giordano, A. , & MacLachlan, M. (2007). Body image in people with lower‐limb amputation: A Rasch analysis of the amputee body image scale. American Journal of Physical Medicine & Rehabilitation, 86, 205–215. 10.1097/PHM.0b013e3180321439 17314705

[nin12351-bib-0024] Gordon, G. S. (2019). The transformation: Discovering wholeness and healing after trauma. New York, NY: HarperCollins.

[nin12351-bib-0025] Griffen, T. C. , Naumann, E. , & Hildebrandt, T. (2018). Mirror exposure therapy for body image disturbances and eating disorders: A review. Clinical Psychology Review, 65, 163–174. 10.1016/j.cpr.2018.08.006 30223161

[nin12351-bib-0026] Jenkinson, P. M. , & Preston, C. (2017). The ‘not‐so‐strange’ body in the mirror: A principal components analysis of direct and mirror self‐observation. Consciousness and Cognition, 48, 262–272. 10.1016/j.concog.2016.12.007 28061429

[nin12351-bib-0027] Keenan, J. P. , Gallup, G. C. , & Falk, D. (2003). The face in the mirror: The search for the origins of consciousness. New York, NY: HarperCollins Publishers.

[nin12351-bib-0028] Levine, M. G. (1986). The subject is taboo. Comparative Literature, 101(5), 977–1002.

[nin12351-bib-0029] Marchetti, A. , Piredda, M. , & De Marinis, M. G. (2016). Centrality of body and embodiment in nursing care: A scoping study of the Italian literature. Journal of Nursing Scholarship, 48(1), 31–28. 10.1111/jnu.12178 26580707

[nin12351-bib-0030] Mason, D. M. (2014). Holism and embodiment in nursing: Using Goethean science to join 2 perspectives on patient care. Holistic Nursing Practice, 28(1), 55–64. 10.1097/HNP.0000000000000010 24304632

[nin12351-bib-0031] Melchior‐Bonnet, S. (2002). The mirror: A history. New York, NY: Routledge.

[nin12351-bib-0032] Merleau‐Ponty, M. (2012). Phenomenology of perception. New York,NY: Routledge.

[nin12351-bib-0033] Morin, A. (2006). Levels of consciousness and self‐awareness: A comparison and integration of various neurocognitive views. Consciousness and Cognition, 15, 358–371. 10.1016/j.concog.2005.09.006 16260154

[nin12351-bib-0034] Ovid . (1955). The metamorphoses (M.M. Innes, Trans.). Harmondsworth, Middlesex, UK: Penguin.

[nin12351-bib-0035] Pendergrast, M. (2003). Mirror mirror: A history of the human love affair with reflection. New York, NY: Basic Books.

[nin12351-bib-0036] Petrocchi, N. , Ottaviani, C. , & Couyoumdjian, A. (2016). Compassion at the mirror: Exposure to a mirror increases the efficacy of a self‐compassion manipulation in enhancing soothing positive effect and heart rate variability. The Journal of Positive Psychology, 12(6), 525–536. 10.1080/17439760.2016.1209544

[nin12351-bib-0037] Piran, N. (2016). Embodied possibilities and disruptions: The emergence of the experience of embodiment construct from qualitative studies with girls and women. Body Image, 18, 43–60. 10.1016/j.bodyim.2016.04.007 27236476

[nin12351-bib-0038] Porges, S. W. (2018). Polyvagal theory: A primer In Porges S. W., & Dana D. (Eds.), Clinical applications of the polyvagal theory: The emergence of polyvagal‐informed therapies (pp. 50–69). New York, NY: W.W. Norton & Company.

[nin12351-bib-0039] Ricoeur, P. (1966). Freedom and nature: The voluntary and the involuntary (E.V. Kohak, Trans.). Evanston, IL: Northwestern University Press.

[nin12351-bib-0040] Shepard, L. , Tattersall, H. , & Buchanan, H. (2014). Looking into the mirror for the first time after facial burns: A retrospective mixed methods study. Burns, 40, 1624–1634. 10.1016/j.burns.2014.03.011 24742782

[nin12351-bib-0041] Stanford Libraries . (n.d.). Snow White and the seven dwarfs. Retrieved January 3, 2020, from https://library.stanford.edu/all/?q=Snow+white+and+the+Seven+dwarfs&op=Search.

[nin12351-bib-0042] Thayer, B. , Freysteinson, W. M. , & Thomas, L. (in press). Transforming the experience of mirror viewing for individuals faced with disfiguring injuries. Clinical Nurse Specialist. 10.1097/NUR.0000000000000518 32250995

[nin12351-bib-0043] Theede, M. (2018). Polyvagal theory affirms the importance of nursing In Porges S. W., & Dana D. (Eds.), Clinical applications of the polyvagal theory: The emergence of polyvagal‐informed therapies. New York, NY: W.W. Norton & Company.

[nin12351-bib-0044] Thieme, H. , Morkisch, N. , Mehrholz, J. , Pohl, M. , Behrens, J. , Borgetto, B. , & Dohle, C. (2018). Mirror therapy for improving motor function after stroke: Update of a Cochrane review. Stroke, 50, e26–e27. 10.1161/STROKEAHA.118.023092 https://doi PMC6513639 29993119

[nin12351-bib-0045] Tiidenberg, K. , & Cruz, E. G. (2015). Selfies, image and the re‐making of the body. Body & Society, 21(4), 77–102. 10.1177/1357034X15592465

[nin12351-bib-0046] van Veluw, S. J. , & Chnace, S. A. (2014). Differentiating between self and others: An ALE meta‐analysis of fMRI studies of self‐recognition and theory of mind. Brain Imaging and Behavior, 8, 24–38. 10.1007/s11682-013-9266-8 24535033

[nin12351-bib-0047] Veale, D. , & Riley, S. (2001). Mirror, mirror on the wall, who is the ugliest of them all? The psychopathology of mirror gazing in body dysmorphic disorder. Behaviour Research and Therapy, 39, 1381–1393. 11758697 10.1016/s0005-7967(00)00102-9

[nin12351-bib-0048] Wicklund, R. A. (1975). Objective self‐awareness. Advances in Experimental Social Psychology, 8, 233–275.

[nin12351-bib-0049] Wilhelm, S. , Greenberg, J. , Rosenfield, E. , Kasarskis, O. , & Blashill, A. (2016). The body dysmorphic disorder symptoms scale: Development and preliminary validation of a self‐report scale of symptom specific dysfunction. Body Image, 17, 82–87. 10.1016/j.bodyim.2016.02.006 26971118 PMC4877234

